# Supplementary material for: Asymmetric metasurface photodetectors for single-shot quantitative phase imaging
Source: Nanophotonics. 2023 Aug 2;12(17):3519–28. doi: 10.1515/nanoph-2023-0354 (PMC11614331; doi:10.1515/nanoph-2023-0354)
Supplement: Supplementary file 1 — Supplementary Material Details [file j_nanoph-2023-0354_suppl_001.pdf]

Supplementary Material

Asymmetric metasurface photodetectors for single-shot quantitative phase imaging

Jianing Liu, Hao Wang, Yuyu Li, Lei Tian, and Roberto Paiella

Department of Electrical and Computer Engineering and Photonics Center,

Boston University, 8 Saint Mary’s Street, Boston, MA 02215

Table of content

S1. Reflector design

S2. Experimental comparison with a reference uncoated device

S3. FDTD calculation of the coherent transfer function

S4. Additional phase imaging results

S5. Derivation of the phase reconstruction protocol

S1. Reflector design

The reflector section of the composite metasurface described in the main text [Fig. 2(a)] consists of an array of Au rectangular nanostripes, designed so that their reflection phase varies linearly with their center position with slope $-2\pi n_{SPP}/\lambda$ (here λ = 1550 nm is the operation wavelength and n_{SPP} is the plasmonic effective index). As a result, any incoming surface plasmon polariton (SPP) traveling along the +x direction is scattered by negative-first-order diffraction into radiation propagating away from the sample along the surface normal. At the same time, all other orders of diffraction are suppressed,[S1] so that these SPPs can be efficiently removed from the device surface without any back-reflection into the grating coupler or straight transmission (which may cause interpixel crosstalk in an array). Figure S1 shows the reflection phase and magnitude of an array of identical nanostripes under normal-incidence plane-wave illumination, computed as a function of nanostripe width via FDTD simulations. All the layer thicknesses used in these simulations are the same as in the optimized structure described in the main text. The red dots in Fig. S1 indicate a chosen set of 3 nanostripes with equally spaced reflection-phase values differing by $2\pi/3$. It should be noted that this set includes a nanostripe of zero width (equivalent to a missing nanostripe), which is convenient for simplifying the fabrication process. The desired linear phase profile with negative slope $-2\pi n_{SPP}/\lambda$ can then be implemented with the periodic repetition of these 3 nanostripes, equally spaced at a distance of $\lambda/(3n_{SPP})$ from one another and with progressively decreasing width (i.e., decreasing reflection phase) along the +x direction. Specifically, in the device developed in this work, the interparticle spacing is 477 nm (based on an FDTD estimate of n_{SPP}) and the entire reflector section consists of 3 repetitions of this set of nanostripes.

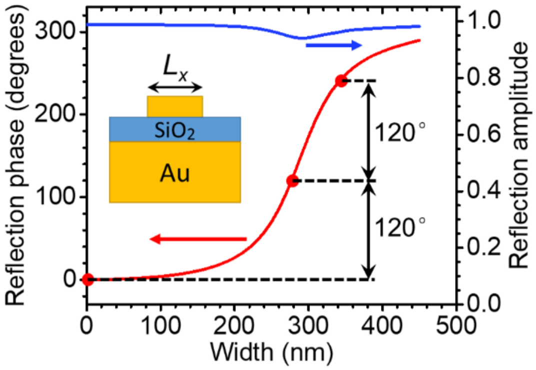

Figure S1. Design of the metasurface reflector section. Reflection phase (red trace) and amplitude squared (blue trace) of the reflector building block (shown in the inset), plotted as a function of nanostripe width for normally incident x-polarized light at λ = 1550 nm. The red circles indicate three nanostripe widths providing equally spaced reflection-phase values differing by $2\pi/3$.

S2. Experimental comparison with a reference uncoated device

The red and blue traces in Fig. S2 show, respectively, the responsivity of the metasurface device described in the main text and of a reference Ge photoconductor, measured as a function of polar illumination angle. The reference device does not include any metasurface on its illumination window, but otherwise was fabricated and measured with the same procedures (described in the Methods section of the main text). In both traces, the responsivity (i.e., output photocurrent per input optical power) is normalized to the device voltage bias. The comparison between these data indicates that, at the angle of peak detection, the metasurface transmission coefficient is about 30%, leading to a proportional reduction in responsivity compared to the reference uncoated device. This value is smaller than, but generally consistent with, the calculated peak transmission coefficient of 38% [see Fig. 2(b) of the main text]. The discrepancy can be attributed to the finite Au-film roughness in the experimental metasurface device, which can partially scatter the excited SPPs into radiation before they reach the slits. It should also be mentioned that, in prior measurements with similar devices,[S2] large sample-to-sample variations were observed (even in devices based on the same design), possibly caused by fabrication-induced defects affecting the carrier density or promoting current leakage. Additionally, we note that the responsivity values in both traces of Fig. S2 are limited by the large inter-electrode separation in these devices, which results in relatively small photoconductive gain.

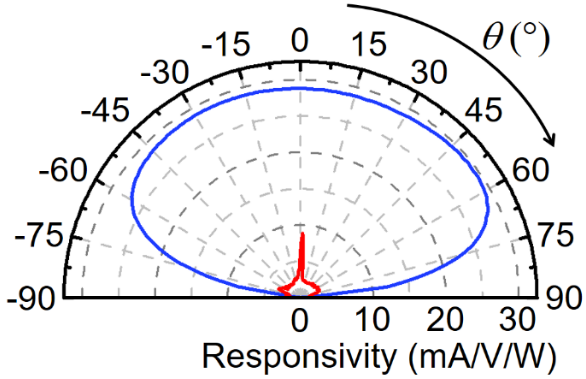

Figure S2. Measured responsivity versus polar illumination angle for the metasurface device described in the main text (red trace) and for an otherwise identical uncoated device (blue trace). The incident light has 1550-nm wavelength and p polarization.

S3. FDTD calculation of the coherent transfer function

To evaluate the phase slope α introduced in eq. (1) of the main text, we used FDTD simulations to compute the coherent transfer function t(**k**) defined in the same equation. In these calculations,

we consider a 2D structure consisting of the entire grating coupler of our experimental sample (without the reflector and slit sections), with perfectly matched layers (PMLs) on all 4 boundaries. The device is illuminated with a p-polarized diffractive plane wave of variable angle of incidence θ. The normal component of the electric field $E_z = |E_z|e^{i\psi}$ (i.e., the dominant SPP field component) is then computed inside the top SiO_2 film far away from the illuminated region as a function of $k_x = (2\pi/\lambda)\sin\theta$. As shown in Fig. S3(a), the resulting plot of $|E_z|^2$ versus k_x is in good qualitative agreement with the device angular response displayed in Fig. 2(b) of the main text. The calculated phase ψ of E_z is plotted as a function of k_x in Fig. S3(b), where the expected linear dependence around the peak location (shaded region) predicted by eq. (1) is clearly observed. The phase slope $\alpha = -d\psi/dk_x$ extrapolated from this plot is about 8 μm.

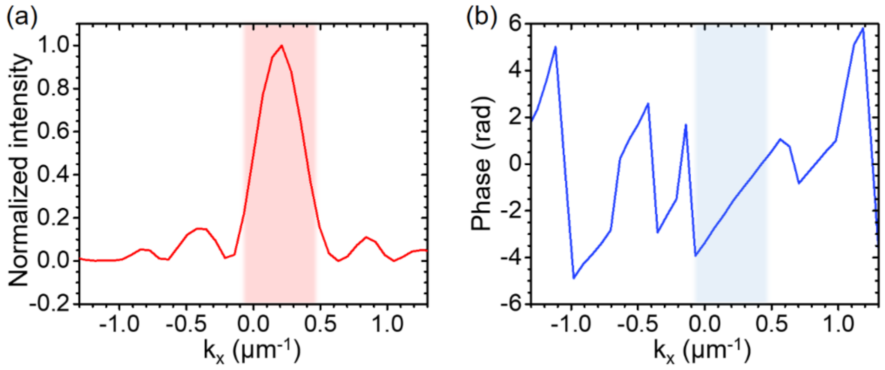

Figure S3. Normalized magnitude (a) and phase (b) of the coherent transfer function $t(k_x,k_y=0)$ of the metasurface device described the main text, computed as a function of k_x via FDTD simulations. The shaded regions in both plots indicate the peak location, where the phase response is approximately linear.

S4. Additional phase imaging results

Figure S4 shows additional phase contrast imaging results for the MCF-10A cell object of Fig. 3(a), constructed with the procedure described in the main text. The image of Fig. S4(a) was computed for an array of uncoated pixels, whose responsivity is essentially constant with incident wavevector (other than the decrease in transmission through the illuminated surface at large angles described by the Fresnel formulas). As expected, the transparent cells could not be visualized with this arrangement, except for negligibly small diffraction fringes. Figures S4(b) and S4(c) show the images computed with the experimental responsivity map of Fig. 2(d), for an array of identical metasurface devices with the nanostripes oriented along the vertical and horizontal directions, respectively [Fig. S4(b) is the same as Fig. 3(b) of the main text]. Finally, Fig. S4(d) shows the phase contrast image recorded with the array configuration of Fig. 5(a), where pixels with

metasurfaces oriented along orthogonal directions are combined in a checkerboard pattern. The green ovals in these plots highlight features of the phase object that are resolved with significantly different contrast depending on the metasurface orientation.

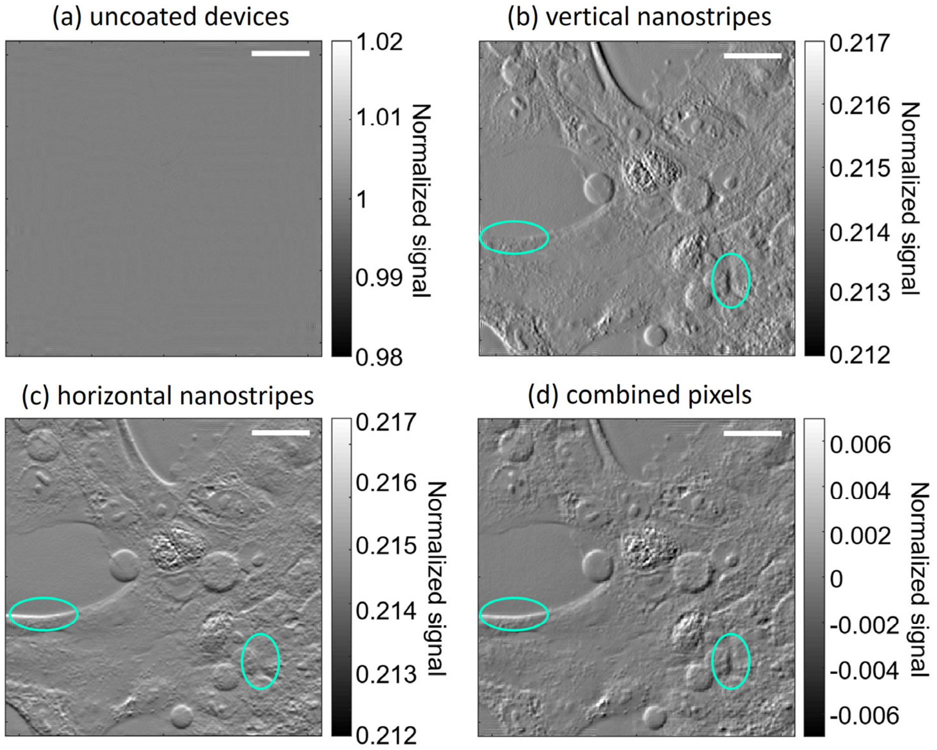

Figure S4. Additional phase contrast imaging results. (a) Image of the phase object of Fig. 3(a) of the main text detected by an array of uncoated pixels. (b) Image of the same object detected with an identical sensor array where each pixel is coated with the metasurface of Fig. 2(d) of the main text. (c) Same as (b) but with the metasurface on each pixel rotated clockwise by 90°. (d) Image of the same object for an array partitioned into blocks of four adjacent pixels, each coated with the metasurface of Fig. 2(d) oriented along one of four orthogonal directions. Specifically, this plot shows the signal $I_{+\hat{\mathbf{x}}}(\mathbf{r}) - I_{-\hat{\mathbf{x}}}(\mathbf{r}) + I_{-\hat{\mathbf{y}}}(\mathbf{r}) - I_{+\hat{\mathbf{y}}}(\mathbf{r})$, as defined in the main text and in section S5. In all four panels, the simulated array consists of 512×512 pixels and is combined with a 40× magnification system with NA = 0.8. The green ovals in (b), (c), and (d) highlight two features of the phase objects that are not clearly resolved depending on the metasurface orientation. The scale bars are 50 μm in all panels.

S5. Derivation of the phase reconstruction protocol

In this section we derive eqs. (3)-(5) of the main text, which provide the basis for the quantitative phase reconstruction method reported in this work. These equations describe the sensor array of Fig. 5(a), which is partitioned into blocks of four adjacent pixels, each coated with the metasurface of Fig. 2 oriented along one of four orthogonal directions ($\hat{\mathbf{u}} = \pm\hat{\mathbf{x}}$ or $\pm\,\hat{\mathbf{y}}$). The phase contrast image recorded by all the pixels with the same metasurface orientation $\hat{\mathbf{u}}$ can be computed as

$$I_{\hat{\mathbf{u}}}(\mathbf{r}) = \iint \frac{d\mathbf{k}}{(2\pi)^2} T_{\hat{\mathbf{u}}}(\mathbf{k}) E_{obj}(\mathbf{k}) e^{i\mathbf{k}\cdot\mathbf{r}} \iint \frac{d\mathbf{k}'}{(2\pi)^2} T^*_{\hat{\mathbf{u}}}(\mathbf{k}') E^*_{obj}(\mathbf{k}') e^{-i\mathbf{k}'\cdot\mathbf{r}}, \quad \text{(S1)}$$

where $E_{obj}(\mathbf{k})$ is the Fourier transform of the incident optical field at the object plane, and $T_{\hat{\mathbf{u}}}(\mathbf{k})$ is a transfer function that accounts for the $\mathbf{k}$-dependence of the pixels and the imaging optics [see eq. (1) of the main text and related discussion]. For a pure phase object, the optical field is $E_{obj}(\mathbf{r}) = \sqrt{P} e^{i\varphi(\mathbf{r})}$, where P and $\varphi(\mathbf{r})$ indicate power and phase profile, respectively. In the weak-object approximation,[S3] $E_{obj}(\mathbf{r})$ can be linearized with respect to $\varphi(\mathbf{r})$, and its Fourier transform becomes

$$E_{obj}(\mathbf{k}) \approx \sqrt{P}[(2\pi)^2 \delta(\mathbf{k}) + i\varphi(\mathbf{k})], \quad \text{(S2)}$$

where $\delta(\mathbf{k})$ is the delta function and $\varphi(\mathbf{k})$ is the Fourier transform of $\varphi(\mathbf{r})$. By substituting eq. (S2) into eq. (S1) and neglecting the term proportional to $\varphi(\mathbf{k})\varphi(\mathbf{k}')$, we find

$$I_{\hat{\mathbf{u}}}(\mathbf{r}) = P\left\{|T_{\hat{\mathbf{u}}}(0)|^2 + i \iint \frac{d\mathbf{k}}{(2\pi)^2} [T^*_{\hat{\mathbf{u}}}(0) T_{\hat{\mathbf{u}}}(\mathbf{k}) - T_{\hat{\mathbf{u}}}(0) T^*_{\hat{\mathbf{u}}}(-\mathbf{k})] \varphi(\mathbf{k}) e^{i\mathbf{k}\cdot\mathbf{r}}\right\}, \quad \text{(S3)}$$

where we have used $\varphi(\mathbf{k}) = \varphi^*(-\mathbf{k})$ since $\varphi(\mathbf{r})$ is real.

From eq. (1) of the main text and its related discussion, the transfer function $T_{\hat{\mathbf{u}}}(\mathbf{k})$ for $\hat{\mathbf{u}} = \pm\hat{\mathbf{x}}$ can be expressed as

$$T_{\pm\hat{\mathbf{x}}}(\mathbf{k}) = C_0 e^{\mp i\alpha k_x} \sqrt{\mathcal{R}(\pm k_x, k_y)}\, t_{lens}(\mathbf{k}), \quad \text{(S4)}$$

where $t_{lens}(\mathbf{k})$ is the contribution from the imaging optics in front of the sensor array, $\mathcal{R}(\mathbf{k})$ is the pixel responsivity map for $\hat{\mathbf{u}} = +\hat{\mathbf{x}}$ [see Fig. 2], α is the phase slope computed in section S3, and C_0 is a normalization factor. Using this expression, eq. (S3) for $\hat{\mathbf{u}} = \pm\hat{\mathbf{x}}$ becomes

$$I_{\pm\hat{\mathbf{x}}}(\mathbf{r}) = |C_0|^2 \mathcal{R}(0) P\left\{1 + \iint \frac{d\mathbf{k}}{(2\pi)^2} H_x(\pm k_x, k_y) \varphi(\mathbf{k}) e^{i\mathbf{k}\cdot\mathbf{r}}\right\}, \quad \text{(S5)}$$

with $H_x(\mathbf{k})$ defined as in eq. (4) of the main text, i.e.,

$$H_x(\mathbf{k}) = i t_{lens}(\mathbf{k}) \left\{\sqrt{\frac{\mathcal{R}(\mathbf{k})}{\mathcal{R}(0)}} - \sqrt{\frac{\mathcal{R}(-\mathbf{k})}{\mathcal{R}(0)}}\right\}. \quad \text{(S6)}$$

In deriving eqs. (S5) and (S6), we have used the fact that $t_{lens}(\mathbf{k})$ is real, even, and unity at $\mathbf{k} = 0$; furthermore, we have neglected the phase term αk_x of eq. (S4), which is appropriate for objects with sufficiently slowly varying phase profile. Using eqs. (S5) and (S6) and the symmetry property $H_x(k_x, k_y) = -H_x(-k_x, k_y)$, we can finally compute the normalized image $S_x(\mathbf{r})$ as follows:

$$S_x(\mathbf{r}) \equiv \frac{I_{+\hat{\mathbf{x}}}(\mathbf{r}) - I_{-\hat{\mathbf{x}}}(\mathbf{r})}{I_{+\hat{\mathbf{x}}}(\mathbf{r}) + I_{-\hat{\mathbf{x}}}(\mathbf{r})} = \iint \frac{d\mathbf{k}}{(2\pi)^2} H_x(\mathbf{k}) \varphi(\mathbf{k}) e^{i\mathbf{k}\cdot\mathbf{r}}. \quad \text{(S7)}$$

Equation (3) of the main text [i.e., $S_x(\mathbf{k}) = H_x(\mathbf{k})\varphi(\mathbf{k})$] is equivalent to the Fourier transform of this equation. The same derivation can be used to evaluate $H_y(\mathbf{k})$ and $S_y(\mathbf{r})$, leading to the same expressions of eqs. (S6) and (S7) with the responsivity map $\mathcal{R}(\mathbf{k})$ rotated by 90°.

The object phase distribution $\varphi(\mathbf{r})$ can then be reconstructed from the measured phase contrast images $I_{\pm\hat{x}}(\mathbf{r})$ and $I_{\pm\hat{y}}(\mathbf{r})$ by solving eq. (S7) and its counterpart with x → y. To avoid numerical issues associated with noise amplification at the zeros of $H_x(\mathbf{k})$ and $H_y(\mathbf{k})$, instead of direct inversion we use the following regularized least-squares minimization

$$\min \sum_{u=x,y} |S_u(\mathbf{k}) - H_u(\mathbf{k})\varphi(\mathbf{k})|^2 + \alpha_T\, |\varphi(\mathbf{k})|^2, \tag{S8}$$

where a_T is a regularization parameter. The solution of this minimization problem is known as Tikhonov regularization[S3] and is given as follows:

$$\varphi(\mathbf{r}) = \mathcal{F}^{-1}\left\{\frac{\sum_{u=x,y} H_u^*(\mathbf{k}) S_u(\mathbf{k})}{\sum_{u=x,y} |H_u(\mathbf{k})|^2 + \alpha_T}\right\}, \tag{S9}$$

which is the same as eq. (5) of the main text.

REFERENCES

S1. F. Ding, A Pors, and S. I. Bozhevolnyi, "Gradient Metasurfaces: a Review of Fundamentals and Applications," *Rep. Prog. Phys.* 81, 026401 (2018).

S2. L. Kogos, Y. Li, J. Liu, Y. Li, L. Tian, and R. Paiella, "Plasmonic Ommatidia for Lensless Compound-Eye Vision," *Nat. Commun.* 11, 1637 (2020).

S3. L. Tian and L. Waller, "Quantitative differential phase contrast imaging in an LED array microscope," *Opt. Express* 23, 11394−403 (2015).
